# Hybridization order is not the driving factor behind biases in duplicate gene losses among the hexaploid Solanaceae

**DOI:** 10.1098/rspb.2022.1810

**Published:** 2022-10-26

**Authors:** Logan McRae, Aleksandra Beric, Gavin C. Conant

**Affiliations:** ^1^ Department of Biological Sciences, North Carolina State University, Raleigh, NC 27695, USA; ^2^ Program in Genetics, North Carolina State University, Raleigh, NC 27695, USA; ^3^ Bioinformatics Research Center, North Carolina State University, Raleigh, NC 27695, USA; ^4^ Department of Psychiatry, Washington University in Saint Louis School of Medicine, St. Louis, MO 63110, USA; ^5^ NeuroGenomics and Informatics Center, Washington University in Saint Louis School of Medicine, St. Louis, MO 63108, USA

**Keywords:** Solanaceae, polyploidy, biased fractionation, hexaploidy, repetitive elements

## Abstract

We model the post-hexaploidy evolution of four genomes from the Solanaceae, a group of flowering plants comprising tomatoes, potatoes and their relatives. The hexaploidy that these genomes descend from occurred through two sequential allopolyploidy events and was marked by the unequal losses of duplicated genes from the different progenitor subgenomes. In contrast with the hexaploid Brassiceae (broccoli and its relatives), where the subgenome with the most surviving genes arrived last in the hexaploidy, among the Solanaceae the most preserved subgenome descends from one of the original two tetraploid progenitors. In fact, the last-arriving subgenome in these plants actually has the fewest surviving genes in the modern genomes. We explore whether the distribution of repetitive elements (REs) in these genomes can explain the biases in gene losses, but while the signals we find are broadly consistent with a role for high RE density in driving gene losses, the REs turn over so quickly that little signal of the RE condition at the time of paleopolyploidy is extant in the modern genomes.

## 1. Introduction

### (a) Polyploidy and genome evolution

The existence of eukaryotes with more than two copies of the genome in their nuclei has long been known [1–4], and prescient suggestions as to the evolutionary importance of such polyploidy events were made throughout the twentieth century [4–6]. However, in an era before the availability of complete genomic sequences, our understanding of how genomes evolved after polyploidy was necessarily limited. For instance, population genetic arguments strongly suggested that the ‘fully duplicated’ state, where every ancestral gene was present in two copies, should be evolutionarily unstable [6–8]. But it was only with the cataloguing of the complete gene complement of a polyploid organism that this loss process could be directly measured [9]. Complete genome sequences have also dramatically expanded the timescale at which polyploidies can be detected, leading to the discovery or confirmation of numerous ancient polyploidy events among flowering plants, vertebrates and other animals [10].

Dense genome sampling from species of yeast with and without their ancient polyploidy allowed for the first careful studies of the timing and distribution of gene losses after polyploidy [11–14]. These analyses demonstrated that the two ‘subgenomes’ created by the yeast whole-genome duplication (WGD) had lost genes in roughly equal fractions. However, as other ancient polyploidy events were studied [15–18], it became evident that the yeast event was unusual in this respect. In fact, of the paleopolyploidies we have so far analysed with our tool the Polyploid Orthology Inference Tool (POInT) [19], only the yeast WGD shows such a balance: in every other case, one of the subgenomes is favoured and has lost many fewer genes than have the others. This imbalance in losses is termed *biased fractionation* [20].

A polyploid genome can form either through the merging of two genomes of the same species (autopolyploidy) or the merger through hybridization of genomes from distinct species (allopolyploidy) [2,3,21]. A pattern of biased fractionation between the resulting subgenomes is more explicable in the case of an allopolyploidy [18], since it is difficult to understand how an autopolyploidy, where the two subgenomes are identical, would generate such biases. However, allopolyploidy need not generate biases either, with the yeast WGD being an allopolyploidy without biased fractionation [22,23].

### (b) Hexaploidy, biased fractionation and the Solanaceae

One of the more interesting paleopolyploidy events in the flowering plants is that shared by tomatoes and their relatives in the family Solanaceae. Even prior to the publication of the first genomes from this family, authors studying genetic maps and expressed sequence tags had proposed the presence of a paleopolyploidy shared by tomatoes and potatoes [24–27]. The completion of the tomato genome made it clear that this event was in fact a hexaploidy [28]. Hexaploid genomes are interesting because there is no known mechanism for generating them in a single mutational step. As a result, they are presumed to form when a tetraploid intermediate undergoes a further polyploidy by hybridization with another diploid [15,29] to yield a triplicated state relative to the ancestor. There is hence, an order to the arrival of the subgenomes for hexaploids that is potentially inferable.

Hexaploid genomes also show biases in their gene losses. Our analysis of the Brassiceae hexaploidy confirmed that one of the three subgenomes retained many more homoeologous genes than did the other two, and we determined that this ‘least fractionated’ (LF) subgenome was the last arriving of the three present [30]. The ‘order of arrival’ effect, therefore, is a potential source of the biased fractionation in hexaploids and makes them a useful system for testing hypotheses about its origins. There are, in essence, three currently proposed drivers of biased fractionation, with the order of subgenome arrival being the first. The second is that a ‘bloom’ of transposable elements (TEs) from one of the subgenomes spread to the other subgenome(s) after the polyploidy, overwriting genes in those subgenomes [31]. The third hypothesis, which is very much the consensus at the moment [32], is that the ancestral subgenomes with high TE density prior to hybridization experienced a global repression of their gene expression after polyploidy and hence suffered more gene losses [32–34]. This idea is mechanistically plausible and consistent with other data. For instance, recent polyploidies show reduced expression from their less-favoured subgenomes, and genes of lower expression should be more prone to homoeologue loss over evolutionary time [34–38]. Since it is also known that the mechanisms by which the host genome silences TEs can reduce the expression of genes in the immediate neighbourhood of those TEs [39], a quite convincing model can be made whereby a higher TE load in a subgenome will drive its average expression level down and, as a longer term consequence, reduce the chances that its encoded genes survive. Interestingly, in many cases, the subgenome with fewest losses in extant polyploid genomes is indeed the one with the lowest TE density [34,35,37,38,40].

However, the current data are incomplete in a few respects. Both a recent transposon bloom and a high ancestral TE load could give rise to a high TE density in fractionated subgenomes. Moreover, TE element turnover is rapid in evolutionary terms [41], raising the question of how reflective of the ancestral condition the modern genomes are. In theory, a phylogenetically informed analysis of multiple genomes sharing a hexaploidy could disentangle these various possibilities.

Here, we use POInT to show that the Solanaceae hexaploidy differs from that in the Brassiceae in that the last-arriving subgenome is in fact the most fractionated; the subgenome with the most surviving genes was instead one of the two arriving in the initial tetraploidy event. We find that the data do not speak clearly as to the role of TE load in driving fractionation because little signal of the ancestral TE distribution survives into the modern genomes. However, to the extent that a pattern is discernible, it is consistent with ancient repetitive element (RE) density being inversely associated with the number of surviving genes from a subgenome.

## 2. Results

### (a) Reconstruction of triple-conserved synteny regions from the Solanaceae hexaploidy

Using our previously described approach [22,30], we inferred a set of triple-conserved synteny (TCS) blocks shared by *Solanum lycopersicum* (tomato)*, Solanum melongena* (eggplant), *Capsicum annuum* (pepper) and *Petunia axillaris*. These blocks consist of 6919 ancestral ‘pillars’ triplicated through hexaploidy; they include 34 148 gene models from across the four genomes. This computation can be thought of as roughly analogous to the multiple alignment step of a phylogenetic analysis. The resulting data are then used as the basis for the POInT analyses that infer orthology between the chromosomal segments of the four extant genomes and make the assignments of genes into subgenomes (see below). Notably, only six of these pillars show the retention of all three gene copies in each of the four genomes, whereas our previous analysis of the Brassiceae hexaploidy found that 418 of 14 050 pillars were fully retained.

### (b) Modelling homoeologous gene loss after hexaploidy

**Figure 1.**
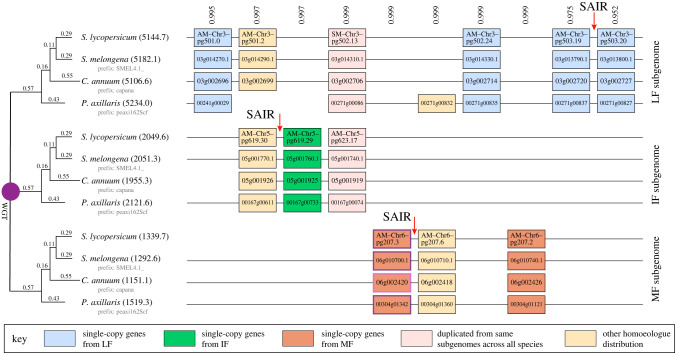
Subgenome assignment and inference of gene loss after the shared hexaploid in four Solanaceae species, showing our inferences of an LF subgenome and two more fractionated ones (IF and MF). The tree at the left illustrates POInT's estimates of the homoeologue loss rates at each stage of the evolution of these four hexaploid genomes. POInT's estimated branch length (*α*t) for each branch is shown above it. This length is used by the model to compute the probability of a gene that is triplicated at the start of the branch experiencing one or more gene losses along that branch. In parentheses, after each taxa are the extant number of genes that POInT estimates survive from that subgenome in that particular modern genome. Shown at the right is an example window of 16 loci produced by whole genome triplication (WGT). Each column (or pillar) corresponds to an ancestral locus triplicated through the hexaploidy, with the boxes representing extant genes. Pairs of genes are connected by lines if they are genomic neighbours in the modern genome. The numbers above each pillar are the posterior probabilities assigned to these combinations of orthology relationships related to the other 6^4^-1 possible orthology states. Three subgenome-assigned intergenic regions (SAIRs) are illustrated (see Methods). (Online version in colour.)

POInT models the loss of homoeologous genes after polyploidy along a phylogeny with a discrete-character maximum-likelihood model similar to that described by Lewis [42]. Because the orthology between the syntenic regions in each genome is not known *a priori*, POInT computes the likelihood of every possible orthology relationship (and thus subgenome assignment) at each pillar and then uses a hidden Markov model (HMM) to condition the orthology inferences at each pillar on those to the left and right [19]. Because for each genome there are 3!=6 possible subgenome arrangements, there are 6^n^ = 1296 possible orthology relationships at each pillar for these *n* = 4 genomes. Figure 1 shows POInT's regional orthology inferences, including the estimated confidence in those orthology relationships and subgenome assignments relative to the other 1296-1 relationships. All of these inferences are also available through POInT_browse_ (wgd.statgen.ncsu.edu).

**Figure 2.**
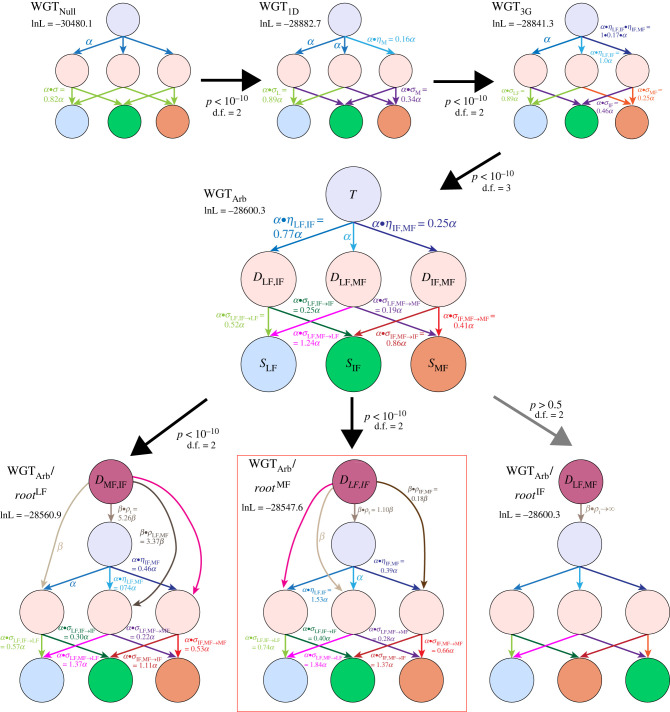
Nested models of hexaploid evolution, showing the loss of duplicated material from the triplicated state (*T*) first to duplicated states (D_X,Y_, where X and Y are the subgenomes where the ancestral gene survives) and then to single-copy states (S_X_, where X is the subgenome preserving the ancestral gene). Arrows depict possible loss events, with the parameters giving the relative rate of loss from that state. All rates are relative to the base loss rate *α*. Using the pillars of TCS (figure 1), we model the losses in those pillars with a series of increasingly complex models of gene loss. WGT_Null_ treats all three of the subgenomes as equivalent: allowing one of the three subgenomes to experience fewer losses (WGT_1D_) significantly improves the fit to the data (*p <* 10^−10^; likelihood ratio test with two d.f.). Similarly, using the WGT_3G_ model to distinguish the other two subgenomes, those of intermediate and most fractionation also improves the fit (*p <* 10^−10^; likelihood ratio test with two d.f.). Because the parameters of this WGT_3G_ model are slightly odd (see Results), we also fit the WGT_Arb_ model, which allows every possible transition between states to occur at an independent rate. This model is an improvement over WGT_3G_ (*p <* 10^−10^; likelihood ratio test with three d.f.). Finally, we tested different models of hexaploid formation, positing models with each subgenome being the ‘last arriving’. In such models, the other two subgenomes formed the initial tetraploid and are shown as duplicated states at the top of each diagram. Hence, if LF is last arriving, all pillars start in state D_IF,MF_, corresponding to IF and MF having formed in the initial tetraploid. Of these three models, that with IF arriving last collapses into the WGT_Arb_ model. Both the LF and MF model improve the fit (*p <* 10^−10^; likelihood ratio test with two d.f. in both cases). However, it is the MF late arrival model that has the highest ln-likelihood and that is consistent with a model where the WGT_Arb_ model is allowed to have differing parameters on the root branch compared to the others (Results*,* electronic supplementary material, figure S2). (Online version in colour.)

The gene loss models can vary in their complexity, with the simplest one having the rate of loss from the triplicated state be the same for all three subgenomes, meaning transitions from the triplicated state T to the three duplicated states (D_X,Y_) occur at the same rate (figure 2). Losses from the duplicated states to the single-copy ones (S_x_ in figure 2) are similarly balanced. We refer to this model as WGT_Null_. Notice that with this model, we cannot assign genes to subgenomes, as all three subgenomes are equivalent in their gene loss rates and statistically indistinguishable.

We can add complexity to the model by allowing one of the three subgenomes (LF) to experience fewer losses than do the other two, creating a model we refer to as WGT_1D_. We find that the WGT_1D_ model fits our pillar data significantly better than does WGT_Null_ (*p <* 10^−10^; figure 2), showing that at least one of the three subgenomes is marked by a lower rate of losses after polyploidy. (Recall that POInT infers the identity of the subgenomes from the pillar data via maximum likelihood.) We next proposed a model allowing the two remaining subgenomes to have differing loss rates as well, namely the WGT_3G_ model (figure 2). Again, this model fits our data significantly better than does WGT_1D_ (*p <* 10^−10^; figure 2), showing that all three subgenomes have differing loss rates. In contrast with our prior nomenclature of MF1 and MF2 for the two more fractionated subgenomes, here we will use the abbreviation ‘IF’ for the subgenome of intermediate fractionation and ‘MF’ for the most fractionated subgenome (figure 1).

The parameter values we infer when fitting the WGT_3G_ model to our data are somewhat difficult to interpret, with the formation of duplicates from LF and either IF or MF (D_LF,IF_ and D_LF,MF_, respectively) apparently occurring at equal rates. By contrast, in the Brassiceae hexaploidy, IF was favoured over MF both in the formation of duplicated and single-copy genes (electronic supplementary material, figure S1). We sought to resolve this confusion with a new ‘arbitrary’ model of hexaploid evolution where each of the nine possible transitions in the model could occur at an independent rate (WGT_Arb_, figure 2). This model fits our data better than did WGT_3G_ (*p <* 10^−10^; figure 2) and more clearly distinguishes the IF and MF subgenomes.

Using this WGT_Arb_ model, we can therefore infer the number of genes from each subgenome surviving in the modern genomes and probabilistically assign individual genes to those subgenomes based on the model's confidence in the regional subgenome assignments (figure 1). Across the four genomes, we infer the number of surviving genes from the MF subgenome to be less than a third of the number surviving from LF, with IF falling in between, with slightly fewer than twice as many surviving genes as MF (figure 1).

### (c) Order of subgenome arrival

The models employed so far do not directly address the question of the order of arrival of the subgenomes. To explore that question, we used our prior approach [30] of comparing three ‘arrival’ models, each proposing a different last-arriving subgenome. The model proposing that IF arrived last collapses back into the WGT_Arb_ model and can be dismissed (*p >* 0.5). However, both the model that proposes that MF was last arriving and the one proposing that LF was last arriving fit the data better than does WGT_Arb_ (*p <* 10^−10^ in both cases; figure 2). Between these two models, which are of equivalent complexity, the model having MF arrive last has the higher likelihood (figure 2) and can therefore be preferred over the model with LF arriving last. For completeness, we applied these same models to our pillar data from the Brassiceae hexaploidy to see if our conclusion that LF was the last-arriving subgenome for that triplication changed with the more complex models used here. However, using the WGT_Arb_ model coupled to the three arrival models still supports LF being the last-arriving subgenome for the Brassiceae hexaploidy (electronic supplementary material, figure S1).

Given that both the models with LF and MF being the last-arriving subgenome fit the pillar data significantly better than does WGT_Arb_, one might be concerned that our data are not sufficient to distinguish between these two arrival models. However, we can also fit a version of the WGT_Arb_ model that allows the root branch to have different values of the model parameters than do the remaining branches. The results of using this model are shown in electronic supplementary material, figure S2. Were the MF subgenome the last arriving, we would expect that transitions from the triplicated state to states preserving this subgenome would be more favoured on the root branch than on the later ones, because MF was present for less of the time represented by that root branch and hence, would have had less time to experience a gene loss than would LF or IF. This pattern is indeed what we observe, with the model favouring transitions from T to D_LF,MF_ over those to D_LF,IF_ on the root branch, but with this preference reversing on the later branches (electronic supplementary material, figure S2). To explain this pattern in a scenario where LF is last arriving, we would be forced to posit that MF was favoured over IF during the tetraploid phase but that this preference switched after the arrival of LF. While not impossible, this explanation is less parsimonious than MF being generally least favoured in fractionation terms over the entire history of these subgenomes.

### (d) Conserved intergenic regions show higher densities of ancestral repetitive elements in the subgenomes of intermediate and most fractionation

To explore the distribution of REs in the different subgenomes, we used POInT's orthology inferences to identify subgenome-assigned intergenic regions (SAIRs). These regions are identified by first finding a pair of neighbouring pillars in the POInT data where one of the three subgenomes has genes present in all four species for both pillars and where each of those pairs of genes from each of four extant genomes are syntenic neighbours (illustrated in figure 1). We then filter these pillar pairs on orthology confidence, retaining only those with confidence greater than or equal to 95%. The intergenic regions between these pairs of high-confidence pillars are then the SAIRs: in other words, orthologous intergenic regions where we know their subgenome of origin with high confidence in all four species.

**Figure 3.**
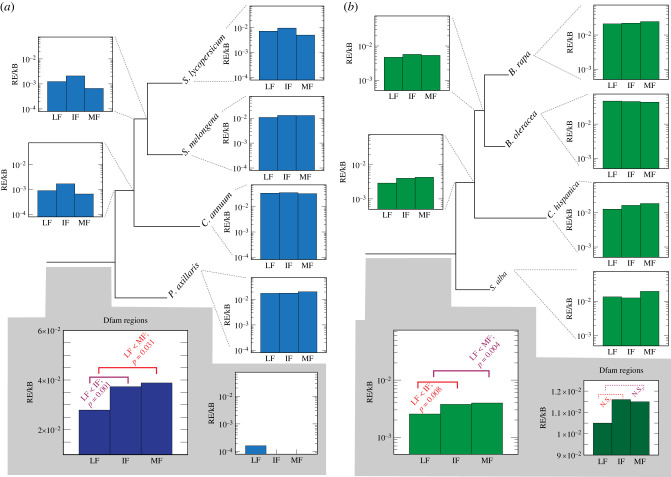
Independent and shared RE density in SAIRs. For each extant genome at the phylogeny tips (right of the trees), we show the repeat density (RE/kB) for each subgenome, inferred with BLASTN and RepBase (Methods). At the phylogeny roots (grey shaded regions) and internal branches (left of the tree), we show the density of repeats that are found in orthologous SAIRs for the species in that clade (e.g. for the root branch, all four species in the tree). Note the log-scale on the *y*-axis of all diagrams except that for Dfam, reporting RE hits per kilobase (kB) of SAIR sequence. For the Brassiceae, there are significantly fewer such insertions shared between all species in the LF genome (see Methods), but this difference is not seen for the Solanaceae genomes. For the Solanaceae, LF is significantly deficient in shared REs when the larger Dfam set of elements is used as a query, but the differences, while in the same direction for the Brassiceae, are non-significant (Methods). (Online version in colour.)

We first used BLASTN [43] to search these regions against the set of green plant REs from RepBase v. 27 [44]. As figure 3 shows, there is a good deal of variability in the RE distribution between the individual genomes, with no clear pattern as to which subgenome has the highest RE density. However, it is also clear that nearly all of the REs in question are recent: there is often an order of magnitude difference in the density of REs found in an individual genome compared to the density of RE insertions that are shared in orthologous positions across the genomes studied and hence assumed to be ancestral (Methods).

In fact, for the SAIRs shared by the four Solanaceae genomes, only nine RE hits are found to be phylogenetically conserved in orthologous positions across the four genomes, and all nine of these are found in the LF subgenome. This observation is not in itself surprising, because LF has 1733 SAIRs, compared to 329 and 110 for IF and MF, respectively. For the four Brassiceae genomes, 147 conserved RE hits are found, and the LF subgenome has significantly lower RE density than either IF or MF (figure 3; *p =* 0.008 and 0.004, respectively; see Methods). However, closer analysis showed that all but two of the conserved elements identified were actually tRNA genes. When we repeated our search of the Brassiceae genomes with a tRNA database [45], we found that indeed LF had lower tRNA density in conserved positions than did IF or MF (*p =* 0.002 and *p <* 0.001, respectively). LF was also the subgenome with the lowest tRNA density in the individual Brassiceae genomes. However, the Solanaceae genomes again only had seven tRNA genes, all from LF, conserved across the SAIRs from the four genomes, and tRNAs were not universally less frequent in LF across these genomes (data not shown).

Because we identified so few RE positions in the Solanaceae genomes using the RepBase dataset, we broadened our search by scanning against all known REs in the Dfam database using nhmmer (see Methods; [46]). When considering the RE regions identified with nhmmer in the tomato genome that showed strongly conserved local alignments to the other three genomes (Methods), we did find that the LF subgenome showed lower RE density than did IF or MF (figure 3; *p =* 0.001 and 0.0318, respectively). When we conducted the corresponding Dfam search in the Brassiceae, identifying Dfam hits in *Brassica rapa* and aligning those regions to the other three genomes, LF showed the numerically lowest RE density, but there was no statistical difference between the subgenomes in RE density (figure 3; *p >* 0.05).

## 3. Discussion

Having two groups of taxa that have independently acquired a hexaploid state illustrates both commonalities and some surprising differences in the patterns of evolution after those events. Most strikingly, unlike in the Brassiceae [15], the order of subgenome arrival does not appear to have greatly influenced which subgenome was favoured for homoeologue retention among the polyploid Solanaceae. Instead, the current LF subgenome was one of the two founding subgenomes, with the MF subgenome arriving later and experiencing a very rapid loss of its genes, such that today it has the fewest surviving genes in each of the extant genomes (figure 1). Of course, the existence of (single formation step) tetraploidies possessing biased fractionation [17] adequately refutes order-of-arrival as the sole source of biased fractionation. But it is still striking that, even with its later arrival, MF was so disfavoured as to be the subgenome with fewest survivors today.

If arrival order is not driving fractionation biases in the Solanaceae, what is? TEs or repetitive sequences more generally are still strong candidates [31–34]. However, it may be worth taking a slightly wider perspective. The gene expression biases in new allopolyploids [34–38] provide a selective environment favouring the loss of the more lowly expressed homoeologous copies. Any process producing such expression biases might, therefore, in the longer term, give rise to subgenomes with fractionation biases. While the repression of repetitive sequences could certainly drive expression biases [39], other differences in the genomic expression landscape between the progenitor species could as well. Bottani *et al*. [38] have pointed out that genomes of differing sizes will experience differing levels of off-target transcription factor binding. As a result, in a larger genome, either the local affinity of a transcription factor for its regulatory motif or the expression of that transcription factor would need to be higher than what is seen in the smaller genome in order to achieve equivalent expression of the target gene between the two genomes.

Hence, we should probably view the RE repression hypothesis as one of a number of potential differences between progenitor genomes that drive biases first in expression and eventually in losses. From that perspective, the major conclusion of our analysis of REs is in fact a negative one: we cannot easily infer the ancestral RE content of the subgenomes from the REs seen in the modern genomes. This conclusion is in accord with our prior finding of little phylogenetic signal in the RE distributions across many species in the Brassiceae [41]. On this basis, we would argue that the RE density of modern subgenomes is probably irrelevant to the question of whether RE density drove biased fractionation in paleopolyploidies.

We nonetheless do find some evidence of increased ancestral RE density in the IF and MF subgenomes for both the Solanaceae and Brassiceae hexaploidies (figure 3), supporting the RE hypothesis for bias formation. Unfortunately, it is difficult to know how much faith to place in these results. The results in the Brassiceae are entirely driven by the density of tRNA genes and, while this is not true of the Solanaceae results, the Dfam database used to find the conserved RE regions is strongly biased to animals [44], so it is somewhat surprising that more hits were found using it that with the green plant REs from Repbase [47].

The bias in tRNA distribution between subgenomes may also support the need for a broader study of factors affecting expression bias in hybrids. In this view, TEs, base composition, epigenetics and gene density will all alter chromatin state and hence, gene expression [31,38,48]. Understanding these effects should illuminate the behaviour of hybrids more generally, including phenomena such as hybrid vigour and heterosis [49,50]. As a result, studying the repressive effects of RE density in recent polyploids remains a very worthwhile endeavour, especially if such studies are coupled to a broader survey of chromatin dynamics both in the allopolyploid and in its progenitors.

## 4. Methods

### (a) Genomes, homology search and synteny block inference

We analysed four genomes sharing the Solanaceae hexaploidy: *Solanum lycopersicum* (tomato; [28]), *Solanum melongena* (eggplant; [51]), *Capsicum annuum* (hot pepper; [52]) and *Petunia axillaris* [53]. The genome of *Coffea canephora* [54] was used as a nonhexaploid reference. The genomes of *S. lycopersicum, P. axillaris* and *C. canephora* were obtained from CoGe [55] with accession IDs 57947, 54659 and 54651, respectively. Genome data for *S. melongena* and *C. annuum* were obtained from the Sol Genomes Network [56].

For each of the four hexaploidy genomes, we followed a three-step process for identifying the TCS blocks produced by the hexaploidy. First, we conducted a homology search against *C. canephora* using GenomeHistory v 2.0 [57]. GenomeHistory uses BLASTP [43] to search for pairs of homologous protein sequences and then aligns them [58] and computes the synonymous and non-synonymous divergence of their encoding genes using the likelihood approach of Muse and Gaut/Goldman and Yang [59,60]. We required homologous pairs of proteins between each hexaploid genome and *C. canephora* to show a maximum BLAST E-value of 10^−10^ and 70% sequence identity over 80% or more of their length. Any genes whose proteins showed no homologue in the other genome at these stringencies were searched again using GenomeHistory with an increased E-value cutoff of 10^−7^ and 50% identity.

Second, using the pairs of homologous genes identified as just described, we sought to identify the TCS blocks in each of the hexaploid genomes. We have previously described this inference process [22,30]: briefly, for a single gene in *C. canephora,* up to three of the homologous genes in a hexaploid genome are allowed to be the homoeologues of that gene triplicated in the hexaploidy. Using simulated annealing [61], we then seek a combination of a gene order and homoeologous gene assignments that maximizes the number of genes that are each other's genomic neighbours.

Third and finally, we merged these TCS blocks from each genome into a global set of ‘pillars’ consisting of between 1 and 3 genes from each hexaploid genome using the *C. canephora* genes as a reference, resulting in 6919 pillars where every gene in that set had at least one synteny relationship with another gene in the set. We then further optimized the pillar order by performing rearrangements on these inferred blocks. This approach approximates the ancestral order that existed before the whole-genome triplication (WGT) and maximizes the number of genes that are neighbours, yielding a total of 5605 synteny breaks across the four genomes.

### (b) Assessing alternative phylogenetic relationships with POInT

Assuming that *S. lycopersicum* and *S. melongena* are sister taxa, we tested the remaining three possible rooted phylogenetic relationships between these four taxa with the WGT_Arb_ model of homoeologue loss: the topology shown in figure 1 has higher log-likelihood than either one placing *P. axillaris* as sister to the *Solanum* species or the one making *C. annuum* and *P. axillaris* sister to each other (−28 600.3 versus −28 793.9 and −28 814.6, respectively).

### (c) Testing models of post-polyploidy homoeologue losses with POInT

We next fit a series of nested models of homoeolog loss to the pillar data using POInT (figure 2, [62]). Under the simplest of these models (WGT_Null_), we allowed a uniform rate of homoeologue loss from the triplicated state T for each of the three subgenomes, with a potentially different rate of loss *σ* from the three resulting duplicated states (D_X,Y_; figure 2). This model corresponds to the case where the three genomes that formed the hexaploid merged instantaneously and without bias. We then sequentially fit models that further differentiated among the subgenomes, first allowing one to have more surviving homoeologues than the other two (WGT_1D_), then allowing all three subgenomes to differ in their loss propensities (WGT_3G_) and finally, allowing the loss rates from each of the three duplicated states to differ (WGT_Arb_). For comparison, we also fit each of these models to the Brassiceae hexaploidy we had previously analysed [30].

### (d) Fitting models of subgenome arrival order

Because hexaploidies are not unitary events, it would be desirable to understand which two of the three extant subgenomes merged first. We have previously described how POInT can model hexaploid formation by allowing each of the three subgenomes to be the ‘last arriving’ [30], as depicted in figure 2. To assess the quality of these models, we also fit a separate version of the WGT_Arb_ model that allowed the root branch of the polyploidy to show different loss parameters than the other branches (electronic supplementary material, figure S2) [22].

### (e) Identifying orthologous intergenic regions from different subgenomes

We identified pairs of nearby pillars in our ancestral order for which we had both high (greater than or equal to 95%) confidence in the subgenome assignments and for which all four genomes had genes present for a given subgenome in each pillar that were each other's syntenic neighbours. Such conserved SAIRs are those for which we have very high confidence in both the orthology of the regions and in their subgenome of origin (figure 1).

### (f) Identifying transposable elements in the conserved intergenic regions

We used two datasets to identify REs in these SAIR intergenic regions: (i) the green plant repeat set from RepBase v. 27 [44] and (ii) Dfam v 3.5 [47]. We identified matches between the Repbase repeats and the SAIRs of all Solanaceae and Brassiceae genomes using BLASTN [43] with an E-value cutoff of *E* ≤ 10^−5^. We used a parsimony approach to identify REs putatively conserved since the common ancestor of the four Solanaceae genomes studied and similarly for the four Brassiceae genomes. Specifically, we considered an RE as ancestral if a hit to that RE was present in all four orthologous intergenic regions at the specified stringency.

To assess whether the differences in RE abundance between the subgenomes were statistically significant, we used a randomization approach. Taking the set of RE hits to SAIRs in an extant genome, we randomized the order of those hits and then placed them at random in the SAIRs. To make such random placements, we determined the total number of positions *t* in the SAIRs available for insertion by subtracting from each region the length of the RE hit and omitting any SAIRs shorter than the RE hit. We then drew a uniform random number between 0 and *t* to assign placement. For the next RE hit, we omitted all previously randomly assigned regions from the computation of *t*, meaning that two randomly assigned hits could never overlap. We computed the density of REs in each subgenome as the number of hits in that subgenome over the total length of all SAIRs from that subgenome. The difference in the RE densities for the randomized hits was compared to that seen for the real genomes. In the case of the ancestral REs, we used a similar approach, using one of the four genomes (*S. lycopersicum* or *B. rapa*) as a base genome for performing the randomizations.

Because almost all of the conserved REs found among the Brassiceae genomes were actually tRNA genes, we similarly searched the SAIRs against a tRNA database [45] using the same approach to search and randomization.

The Dfam database is much larger than RepBase and allows for more precise RE identification using hidden Markov models. Hence, we took a different approach for identifying REs from Dfam. First, we used nhmmer from the hmmer 3.3.2 package [46] to search the intergenic regions of *S. lycopersicum* and *B. rapa* against the full Dfam HMM collection with an E-value cutoff of less than or equal to 10^−5^. This search is computationally costly and will yield regions where multiple RE HMM models may intersect or overlap. Thus, instead of repeating this search in other genomes, we first assembled RE ‘regions’ by merging any HMM hits to SAIRs that overlapped by at least one DNA base. We next used these RE regions from *S. lycopersicum* or *B. rapa* as queries for local alignment [63] against the orthologous SAIRs from the other three genomes. Our alignment scoring function was matches: +4, mismatches: −5, gap opening penalty: −8, gap extension penalty: −4. Any RE region with a local alignment of at least 80 bases and an alignment score of 200 or more in each of the other three genomes was considered to be an ancestral RE region. Then, we again used the *S. lycopersicum* and *B. rapa* SAIRs as references and applied the same randomization approach as above to assess the significance of any differences in the RE density between the subgenomes.

## Data Availability

All of the data used in this manuscript were previously published and are freely accessible via the accession numbers provided in the text. The POInT software package is available from GitHub: https://github.com/gconant0/POInT/releases/tag/v1.55. All of the underlying inferences, models, phylogenetic trees and gene order sets are freely available from the POInT_browse_ web portal: wgd.statgen.ncsu.edu. Data are also provided in the electronic supplementary material [64].
